# The distinct clinical features and prognosis of the CD10^+^MUM1^+^ and CD10^−^Bcl6^−^MUM1^−^ diffuse large B-cell lymphoma

**DOI:** 10.1038/srep20465

**Published:** 2016-02-09

**Authors:** Ting-Xun Lu, Yi Miao, Jia-Zhu Wu, Qi-Xing Gong, Jin-Hua Liang, Zhen Wang, Li Wang, Lei Fan, Dong Hua, Yao-Yu Chen, Wei Xu, Zhi-Hong Zhang, Jian-Yong Li

**Affiliations:** 1 Department of Hematology, the First Affiliated Hospital of Nanjing Medical University, Jiangsu Province Hospital, Nanjing 210029, China; 2 Department of Oncology, the Affiliated Hospital of Jiangnan University, Wuxi 214062, Jiangsu Province, China; 3 Department of Pathology, the First Affiliated Hospital of Nanjing Medical University, Jiangsu Province Hospital, Nanjing 210029, China; 4 Collaborative Innovation Center For Cancer Personalized Medicine, Nanjing Medical University, Nanjing 210029, China

## Abstract

Using an immunohistochemistry (IHC) based method, diffuse large B-cell lymphoma (DLBCL) can be classified into germinal center B-cell (GCB) and non-GCB subtypes. However, the prognostic value of Hans algorithm was contradictory in the literature. Using IHC and fluorescence *in situ* hybridization, we analyzed the antibodies applied in Hans algorithm and other genetic factors in 601 DLBCL patients and prognostic value of Hans algorithm in 306 cases who were treated with chemoimmunotherapy. The results showed that patients with GCB subtype have better overall survival (OS) and progression-free survival (PFS) than non-GCB cases. However, to some extent, double positive (CD10^+^MUM1^+^, DP) and triple negative (CD10^−^Bcl6^−^MUM^−^, TN) showed different clinical characteristics and prognosis to others that were assigned to the same cell-of-origin group. The DP group showed similar OS (median OS: both not reached, *P* = 0.3650) and PFS (median PFS: 47.0 vs. 32.7 months, *P* = 0.0878) with the non-GCB group while the TN group showed similar OS (median OS: both not reached, *P* = 0.9278) and PFS (median PFS: both not reached, *P* = 0.9420) with the GCB group. In conclusion, Recognition of specific entities in Hans algorithm could help us to accurately predict outcome of the patients and choose the best clinical management for them.

Diffuse large B-cell lymphoma (DLBCL) is the most common lymphoid malignancy in adults, accounting for 30–40% of all non-Hodgkin lymphomas (NHL) in Western countries^1^. The introduction of chemoimmunotherapy in the treatment of DLBCL has dramatically improved the outcome of these patients compared with chemotherapy alone^2^,^3^,^4^. However, a significant proportion of these patients (30% to 40%) become refractory to the treatment or eventually relapse^5^. Therefore, the identification of factors, either biologic or clinical, that can identify patients at a higher risk has priority. International prognostic index (IPI) based on clinical parameters is widely used for risk stratification but might be not reliable to predict outcome in individual patients because of biological diversity^6^. Many biomarkers have been investigated to minimize this residual heterogeneity, but few exhibits sufficient prognostic power^6^.

DLBCLs can be classified by gene expression profiling (GEP) studies into germinal center B cell-like (GCB) group, activated B cell-like (ABC) group and type 3, with the latter two having significantly worse outcome than the GCB subtype^7^. Furthermore, patients with GCB or ABC DLBCLs may benefit from different therapeutic approaches, with the ABC subtype responding to novel drugs (bortezomib, lenalidomide or ibrutinib)^8^. However GEP analysis is not practical in the clinical laboratory. As a result, immunohistochemistry (IHC) algorithms using formalin-fixed, paraffin-embedded (FFPE) tissue have been proposed to predict the GEP subtypes. Among the published IHC algorithms, Hans algorithm was most widely used in routine practice^9^. In addition to the low agreement with GEP data^9^,^10^,^11^,^12^,^13^,^14^, prognostic significance of the Hans algorithm has also been questioned, especially in the chemoimmunotherapy era^8^,^15^,^16^,^17^,^18^.

Hans algorithm was made up of three markers (CD10: GCB marker; Bcl6: associated with both GCB and ABC subtype; MUM1: post GC marker)^8^,^9^. Based on combination of the three markers, Hans algorithm could divide DLBCL into two groups (GCB and non-GCB subtype). Although MUM1 is used as a post GC marker, cases with coexpression of CD10 and MUM1 (CD10^+^MUM1^+^, double positive or DP), which was classified as GCB subtype according to Hans algorithm, do exist. However, the differences of clinical characteristics and prognosis between DP and other GCB (GCB excluding DP) are unknown. Additionally, DLBCL without any positive staining of these three markers (CD10^–^Bcl6^–^MUM1^–^, triple negative or TN) were also noted. These cases, based on Hans algorithm, are classified as non-GCB subtype. However, little is known about the difference between TN cases with GCB or other non-GCB (non-GCB excluding TN).

In this study, we analyzed the clinical features of different groups: GCB vs. non-GCB; DP vs. other GCB (GCB*) or non-GCB; TN vs. other non-GCB (non-GCB*) or GCB. Besides, we further analyzed the survival differences among above groups in patients who were treated with rituximab based chemoimmunotherapy and well followed up.

## Patients and Methods

### Patients

All experimental protocols were approved by the Ethics Committee of the First Affiliated Hospital of Nanjing Medical University and were performed in accordance with the approved guidelines of Nanjing Medical University. All patients provided informed consent in accordance with requirements of the Declaration of Helsinki. We reviewed the medical records of patients who diagnosed as *de novo* DLBCL at our hospital between September 2006 and October 2014. Cases of special variants or subtypes, such as primary central nervous system lymphoma, primary mediastinal B-cell lymphoma and HIV-positive DLBCL were excluded from the whole cohort. Finally, a total of 601 cases were included in the study. Among these, 306 cases were treated with rituximab based chemoimmunotherapy and well followed up. The treatments included R-CHOP (rituximab, cyclophosphamide, doxorubicin, vincristine, prednisone), dose-adjusted (DA)-EPOCH (etoposide, prednisone, vincristine, cyclophosphamide, doxorubicin) plus rituximab and R-CHOP-like regimens included R-CDOP (rituximab, cyclophosphamide, pegylated liposomal doxorubicin, vincristine, prednisone), R-CEOP (rituximab, cyclophosphamide, etoposide, vincristine, prednisone) and R-miniCHOP (rituximab combined with low-dose CHOP). The median follow up time was 24.3 months (1 to 115.4 months).

### IHC

IHC was performed on 4-μm FFPE sections. The antibodies used were CD10 (clone 56C6, Dako), CD20 (clone L26, Abcam), Bcl6 (clone LN22, Dako), and MUM1 (clone MUM1p, Dako), Myc (clone Y69; Abcam, cut-off: 40%) and Bcl2 (clone 124; Dako, cut-off: 50%) (Figure S1). The cut-off scores for each antibody were described previously^9^,^19^. Cases positive for both CD10 and MUM1were classified as DP group. Cases negative for CD10, Bcl6 and MUM1 were defined as TN group. Cases positive for both Myc and Bcl2 or Bcl6 were defined as double expression lymphoma (DEL)^19^.

### Fluorescence *in situ* hybridization

Fluorescence *in situ* hybridization (FISH) was carried out according to manufacturer’s instructions on FFPE tissue sections with the following probes: *MYC* dual-color, break apart translocation probe (Vysis LSI) and *IGH/BCL2* dual-color, and dual fusion translocation probe (Vysis LSI). For cases with *MYC* translocation, *BCL6* dual-color break apart rearrangement probe (Vysis LSI) was further analyzed (Figure S2). For probe signal scoring, a minimum of 200 interphase nuclei were examined. The cut-off levels for the probes were established by evaluating the split signals distribution in samples of reactive lymphoid tissues, calculating the mean number of split signals plus three times the standard deviation. The cut-off levels for positive values (mean of normal control ± 3 SD) were 14%, 5% and 7% for *MYC* break apart probe, *IGH*/*BCL2* dual-fusion probe and *BCL6* break apart probe, respectively. As described before, cases with both *MYC* and *BCL2* or *BCL6* translocations were defined as doube hit lymphoma (DHL)^19^.

### Statistical analysis

Statistical analyses were performed with SPSS software, version 20.0. Response to treatment, overall survival (OS) and progression-free survival (PFS) were defined according to Cheson 2014^20^. Chi-squared and Fisher exact tests were used to determine correlation in the frequencies between groups. The actual survival analysis was performed according to the Kaplan-Meier method, and the curves were compared with the log-rank test. For all tests, a *P* value less than 0.05 was considered statistically significant.

## Results

### Patient characteristics

Medical records were reviewed for clinical information including age, sex, serum lactate dehydrogenase (LDH) level, clinical stages (Ann Arbor stage), Eastern Cooperative Oncology Group performance status (ECOG PS), more than one extranodal sites involvement (ESI), IPI and B symptoms. A total of 601 patients were enrolled in our analysis. The median age of the total patient groups was 58.0 years (range, 18 to 88 years), with 43.1% (259/601) of the patients older than 60 years. There was a male predominance (1.35:1), with 62.3% (374/600) and 33.3% (199/597) of the patients presenting with advanced Ann Anbor stage (III/IV) and high-risk IPI score (>2), respectively.

### The association between protein expression and clinical characteristics

The staining of the antibodies applied in this study was showed in Figrue S1. CD10 expression was detected in 26.8% (161/601) of our patients. CD10 expression was associated with normal LDH level (*P* < 0.0001), localized Ann Arbor stage (I or II) (*P* = 0.0014), better PS (*P* = 0.0087), low-risk IPI (≤2) (*P* = 0.0060) and absence of B symptoms (*P* < 0.0001) (Table 1). We detected Bcl6 staining in 66.9% (402/601) cases, there was no correlation was found between any of these clinical parameters and Bcl6 expression (Table 1). Using a cut-off of 30%, MUM1 expression was detected in 65.9% (396/601) of these patients. MUM1 expression was more frequently presented with elevated LDH level (*P* < 0.0001), advanced Ann Arbor stage (III or IV) (*P* = 0.0001), poor PS (*P* < 0.0001), more than one ESI (*P* = 0.0454), high-risk IPI (*P* < 0.0001) and B symptoms (*P* = 0.0005) (Table 1). Besides, cases without MUM1 expression were enriched for younger patients (*P* = 0.0026) (Table 1).

### The association between different groups and clinical characteristics

Based on the expression of these three markers, 232 and 368 cases were classified as GCB and non-GCB subtype, respectively. Non-GCB patients more often presented with unfavorable clinical variables including age older than 60 (*P* = 0.0432), elevated LDH level (*P* < 0.0001), advanced Ann Arbor stage (III or IV) (*P* < 0.0001), poor PS (*P* < 0.0001), high-risk IPI (*P* < 0.0001) and presence of B symptoms (*P* < 0.0001) than GCB subtypes (Table 2).

The differences in clinical features between the DP and non-GCB or GCB* group were listed in Table 3. The incidence of the DP patients was 13.3% (80/601, 95% CI: 10.7–16.3%). The DP phenotype was more likely to occur in elderly patients (*P* = 0.0432) and associated with poor PS (*P* = 0.0192) than GCB* phenotype. Additionally, although not statistically significant, the DP group showed higher incidence of DHL than GCB* patients (10.7% vs 2.9%, *P* = 0.139). There is a trend that *BCL2* rearrangement in GCB* group is more frequent than that in the DP group, although without significance (*P* = 0.06), suggesting that these two groups may be biologically different. However, the DP group and GCB* group did not differ with regard to any of other clinical variables. By contrast, unfavorable clinical variables including elevated LDH level (*P* = 0.0006), B symptoms (*P* = 0.0006), advanced Ann Arbor stage (III or IV) (*P* = 0.0008), and high-risk IPI (*P* = 0.0076) were less frequently presented in the DP group than in the non-GCB group. In addition to this, the DP group tended to have a better PS (*P* = 0.0681) than the non-GCB group. However, the DP group showed higher incidence of DEL (Myc and Bcl2 coexpression) (*P* = 0.044), *MYC* rearrangement (*P* = 0.016) and DHL (*P* = 0.029) than non-GCB group. As both *MYC* rearrangement and DHL predicted worse outcome in our cohort^21^, the biological features of the DP group suggest this group of patients may have unfavorable prognosis.

The differences of the clinical characteristics between the TN and GCB or non-GCB* group were listed in Table 4. The incidence of the TN patients was 8.8% (53/601, 95% CI: 6.7–11.4%). There was no difference in any of the clinical characteristics between the TN and GCB group. Although without statistical significance, elevated LDH level (*P* = 0.0567), B symptoms (*P* = 0.0582) and DEL (*P* = 0.055) were more common in the non-GCB* group than in the TN group. In addition to this, advanced Ann Arbor stage (III or IV) (*P* = 0.0442), better PS (*P* = 0.0321), high-risk IPI (*P* = 0.0042) as well as Myc expression were significantly more common in the non-GCB* group than the TN group. Further, *BCL2* rearrangement, which was of no prognostic value in DLBCL in our cohort^21^, was more common in TN group. These data raised the possibility that patients in TN group had a better prognosis than patients in non-GCB* group.

### Prognosis analysis

On the basis of above analysis, we could at least speculate that patients in the TN group may have a better prognosis than patients in the non-GCB* group. To extend our finding, we performed survival analysis in a cohort of 306 patients treated with chemoimmunotherapy for whose follow up data were available. The detailed characteristics of these patients were listed in Table 5. We compared the clinical characteristics of these 306 patients with those of the entire cohort, and we found that there was no significant difference between these two groups (both in whole, and DP or TN group) (supplementary material Table S1–2), suggesting that these 306 patients maintained the characteristics determined by the entire initial cohort.

### GCB vs. non-GCB

**Figure 1:**
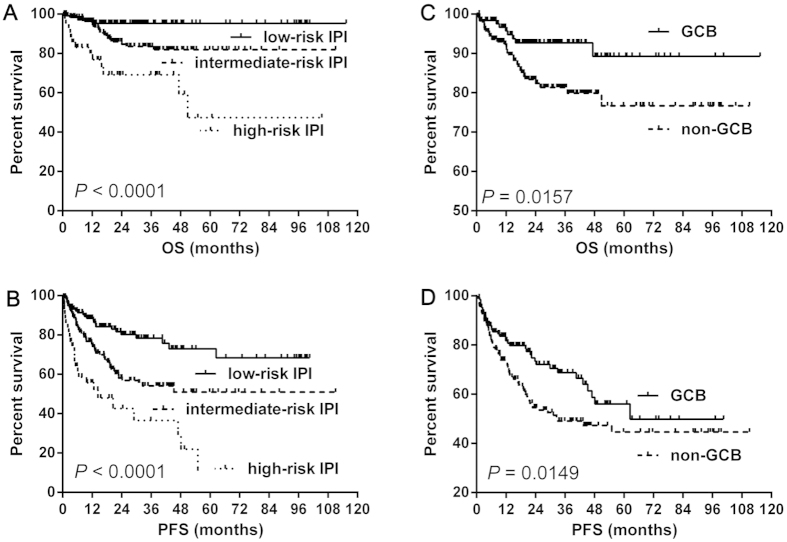
The prognosis of IPI and Hans algorithm. The IPI score stratified patients into three groups with distinct OS (**A**) and PFS (**B**). The Hans algorithm showed significant difference between GCB and non-GCB group (**C,D**). Abbreviations: IPI: International Prognostic Index; GCB: germinal center B-cell; OS: overall survival; PFS: progression-free survival.

In present study, when stratifying our patients into three groups according to IPI (low-risk versus intermediate-risk versus high-risk), and the IPI score predicted OS (*P* < 0.00001) and PFS (*P* < 0.00001) (Fig. 1A,B). The GCB group still had a better OS (median OS: both not reached, *P* = 0.0157) (Fig. 1C) and PFS (median PFS: 62.5 versus 32.7 months, *P* = 0.0149) (Fig. 1D) than the non-GCB group.

### The prognosis of single marker in Hans algorithm

**Figure 2:**
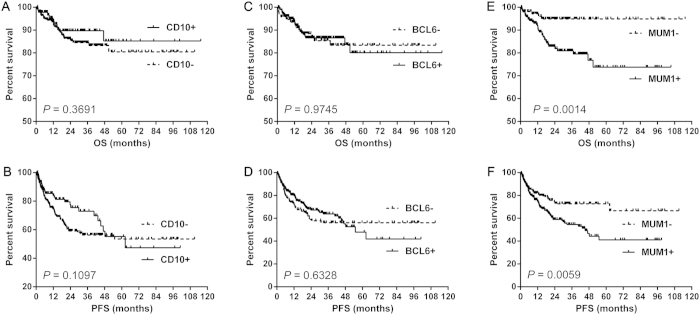
The prognosis of single marker in Hans algorithm. Neither CD10 nor Bcl6 expression was predictive of OS or PFS in DLBCL patients (**A**–**D**). However, MUM1 expression was a significant predictor of worse OS (**E**) and PFS (**F**) in DLBCL patients. Abbreviations: OS: overall survival; PFS: progression-free survival.

With respect to the prognostic value of single marker, neither CD10 nor Bcl6 expression was predictive of OS or PFS (Fig. 2A–D). However, MUM1 expression was a significant predictor of worse OS (median OS: both not reached, *P* = 0.0014) (Fig. 2E) and PFS (median PFS: 45.3 months versus not reached, *P* = 0.0059) (Fig. 2F).

### The DP group vs. GCB* or non-GCB

**Figure 3:**
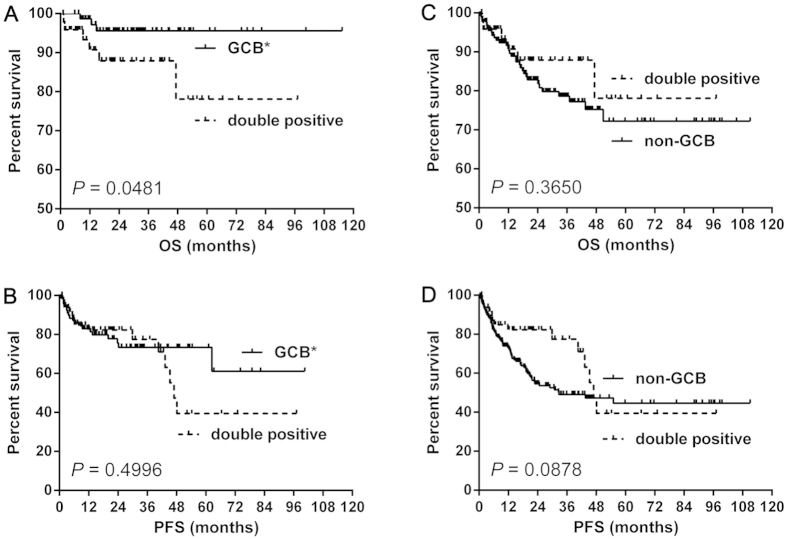
The survival difference between the double positive (CD10^+^MUM1^+^) group and GCB* or non-GCB group. The CD10^+^MUM1^+^ showed a better OS (**A**) but not PFS (**B**) than GCB*. However, the CD10^+^MUM1^+^ showed similar OS (**C**) and PFS (**D**) with non-GCB. Abbreviations: GCB: germinal center B-cell; GCB*: GCB without CD10^+^MUM1^+^ patients; OS: overall survival; PFS: progression-free survival.

The numbers of patients with the DP, GCB* and non-GCB phenotype were 49, 77 and 180, respectively. The patients of the DP group showed a worse OS (median OS: both not reached, *P* = 0.0481) (Fig. 3A) but not PFS (median PFS: 47.0 months vs. not reached, *P* = 0.4996) (Fig. 3B) than those of the GCB* group. However, patients of the DP group had a similar OS (median OS: both not reached, *P* = 0.3650 (Fig. 3C) and PFS (median PFS: 47.0 vs. 32.7 months, *P* = 0.0878) (Fig. 3D) with those of the non-GCB group.

### The TN group vs. non-GCB* or GCB

**Figure 4:**
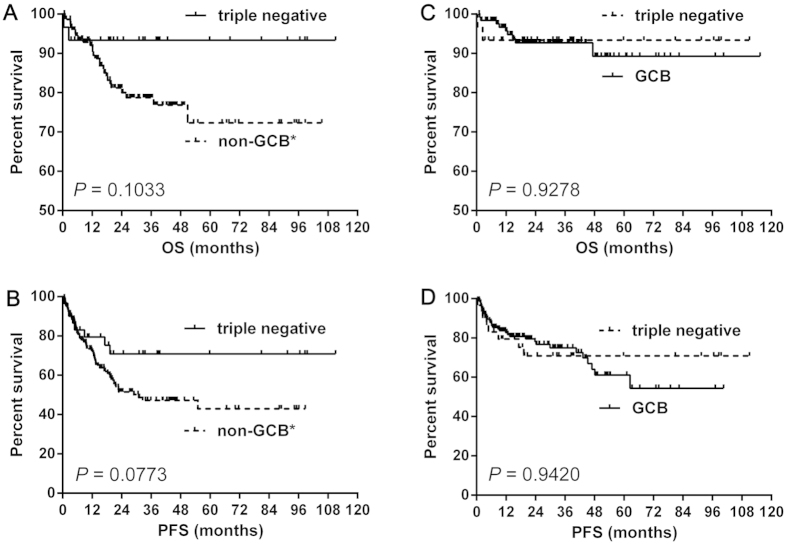
The survival difference between the triple negative (CD10^−^Bcl6^−^MUM1^−^) group and non-GCB* or GCB group. The triple negative group tended to have better OS (**A**) and PFS (**B**) than non-GCB*. However, the triple negative group showed similar OS (**C**) and PFS (**D**) with GCB group. Abbreviations: GCB: germinal center B-cell; non-GCB*: non-GCB without triple negative patients; OS: overall survival; PFS: progression-free survival.

The numbers of patients with the TN, non-GCB* and GCB phenotype were 30, 150 and 126, respectively. The patients of TN group tended to have better OS (median OS: both not reached, *P* = 0.1033) (Fig. 4A) and PFS (median PFS: not reached vs. 30.9 months, *P* = 0.0773) (Fig. 4B) than non-GCB* group. However, patients of the TN group showed similar OS (median OS: both not reached, *P* = 0.9278) (Fig. 4C) and PFS (median PFS: both not reached, *P* = 0.9420) (Fig. 4D) with those of the GCB group.

## Discussion

In the present study, we assessed the clinical features in 601 DLBCL patients and the prognostic value of Hans algorithm in 306 cases who were treated with chemoimmunotherapy. The results showed that GCB DLBCL patients still had better OS and PFS than non-GCB DLBCL patients. Intriguingly, 13.3% (80/601) CD10^+^MUM1^+^ (DP) and 8.8% (53/601) CD10^−^Bcl6^−^MUM1^−^ (TN) DLBCLs were identified in a cohort of 601 patients. Accordingly, the reported incidences of the DP and TN were 2.3–14.3% and 5.5–19.1%, respectively^9^,^22^,^23^. However, TN DLBCLs, which should be classified as non-GCB subtype according to Hans algorithm, were found to have different clinical characteristics from non-GCB* DLBCL. Furthermore, in the analysis of 306 patients with follow-up data, TN phenotype was associated with better OS and PFS compared with non-GCB* phenotypes. Besides, DP DLBCL patients demonstrated worse survival than other GCB patients.

Because of the clinical and biological heterogeneity of DLBCL, much effort has concentrated on identifying effective biomarkers to predict the outcome and aiding the clinical management of patients^9^,^12^,^13^,^14^,^24^. GEP studies have assessed the biology of DLBCL on a molecular basis, which classify DLBCL patients into GCB, ABC and type 3 groups^7^,^25^. The GCB group has a significantly better outcome than the ABC group^9^. However, IHC algorithms were introduced in order to translate the signatures identified by GEP into protein-based tests due to the high cost of microarray analysis and the fresh-frozen tissue requirement^9^,^11^,^13^. However, the majority of such algorithms were developed in the chemotherapy era and their predictive value in patients treated with chemoimmunotherapy was controversial^10^,^11^,^12^,^18^,^26^,^27^. Among these, the most studied one is Hans algorithm, which uses the immunohistochemical staining of CD10, Bcl6, and MUM1 to classify cases of DLBCL into GCB or non-GCB groups^9^.

In the current study, we assessed the clinical relevance and the prognostic value of the single marker in Hans algorithm. Significant associations between clinical features and CD10 and MUM1, but not Bcl6 were observed, which was consistent with previous reports^8^,^28^. In further survival analysis, we found that CD10 expression did not show any prognostic significance, although CD10 expression was associated with favorable clinical variables, which indicated that chemoimmunotherapy employed in our cohort might to some extent overcome the poor prognosis of the group without CD10 expression. However, only MUM1 remained a robust prognostic factor predictive of OS and PFS, which indicated that clinical factors may not always accurately predict disease outcome^29^. We then analyzed the clinical and prognostic values of Hans algorithm. The result showed a significant correlation between non-GCB subtype and unfavorable clinical characteristics. In addition, GCB subtype still demonstrated better OS and PFS than non-GCB, which was consistent with a recent study^29^. Although our study has proved that IHC algorithms retain prognostic significance in the chemoimmunotherapy era, controversy remains in the literature^8^,^15^,^16^,^17^,^18^. This is due most commonly to differences in the patient populations, antibodies and protocols employed^28^ and partly attributed to a lack of homogeneous or sizeable cohorts that are representative of both the population and the clinic^29^. Besides, the existence of some special entities (for example the DP and TN groups) might hinder the prognostic value of Hans algorithm, which was generally neglected in other studies^8^,^17^,^18^.

There is also another reason for us to pay special attention to the DP and TN groups. Bcl6 staining is a technical challenge to perform and the interpretation of samples between pathologists is highly variable^28^, and Bcl6 is not a biomarker with certain definition of the origin of cell^8^. Reports in the chemoimmunotherapy era with Bcl6 expression are inconclusive^30^,^31^. In addition, if the algorithms using Bcl6 (according to Hans or Choi) were modified by its exclusion, they showed similar results as unmodified ones^11^. All of these suggest Bcl6 is an indefinite marker in prediction of outcome and the interpretation of Bcl6 negativity is easier than that of Bcl6 positivity. As a result, we paid close attention to the DP group instead of triple positive and to the TN group rather than single positive of Bcl6 (CD10^−^Bcl6^+^MUM1^−^, defined as GCB subtype by Hans algorithm).

It should be noted that although patients with DP DLBCL did not harbor more unfavorable clinical characteristics than GCB* patients, the DP group predicted worse OS than GCB* patients, which suggested the DP and GCB* groups may respond differently to rituximab based chemoimmunotherapy.

Meanwhile, the TN group showed similar clinical features with GCB but better than non-GCB*. Consistently, the TN group tended to have better OS and PFS than non-GCB* but similar survival to GCB.

The mechanisms underlying our findings remain to be determined. CD10 and Bcl6 are well-defined GC B cell markers, while MUM1, which is a NF-kappa B target gene, is expressed by activated B cells that have the capacity to differentiate towards plasma cells^22^,^32^. MUM1 expression occurs in the final step of B cell in the germinal center (GC). In a previous study, coexpression of at least GC-B cell marker (CD10 or Bcl6) and one of activation markers (MUM1 or CD138) was classified as activated GC B-cell pattern^22^. In the above study, the prevalence of the DP and TN patients were 14.3% (6/42) and 9.5% (4/42), respectively^22^, and patients of activated GC B-cell pattern had similar OS to that of patients expressing at least one activation marker but not GCB markers, which indicated that DP patients might have a relative poor prognosis. In the study of Hans algorithm, the incidences of the DP and TN DLBCLs were 7.2% (11/152) and 19.1% (29/152)^9^ and they showed expression of MUM1 in at least 30% cells predicted worse outcome^9^. In our study, MUM1 expression is also associated with worse OS in CD10^+^ (the DP group) DLBCL patients. The TN DLBCLs remain an ill-defined entity, to our knowledge, there are no available studies regarding the biological and clinical behaviors of these DLBCLs. Based on molecular profiling studies, DLBCLs without both GC B cell and activated B cell gene expression features were termed type 3 group^7^. In chemotherapy era, type 3 patients showed similar OS compared with patients with ABC group. In contrast, type 3 DLBCL patients had similar outcome with GCB group in chemoimmunotherapy era^33^. In our study, patients with the TN group who are treated with chemoimmunotherapy have a prognosis as good as GCB group. It is still unclear whether the TN group exhibits biological and clinical characteristics similar to that of type 3 group. Further studies are needed to investigate the biological and clinical behaviors of the TN group.

In summary, although Hans algorithm remains its prognostic value in current study, cases in the DP and TN groups might hinder the intrinsic power of Hans algorithm in other studies. More detailed classification of DLBCL based on Hans algorithm may help to identify patients with distinct clinical characteristics and prognosis, thereby improving the stratification of patients for risk-adjusted therapies.

## Additional Information

**How to cite this article**: Lu, T.-X. *et al.* The distinct clinical features and prognosis of the CD10^+^MUM1^+^ and CD10^−^Bcl6^−^MUM1^−^ diffuse large B-cell lymphoma. *Sci. Rep.* **6**, 20465; doi: 10.1038/srep20465 (2016).

## Supplementary Material

Supplementary Information

## Figures and Tables

**Table 1:** The association between protein expression and clinical characteristics.

	Older age (>60)	Male predominance	Elevated LDH	Stage III/IV	ECOG PS > 1	ESI > 1	IPI > 2	B symptoms
CD10	*P* = 0.3167	*P* = 0.6520	***P*** < **0.0001**	***P*** = **0.0014**	***P*** = **0.0087**	*P* = 0.7710	***P*** = **0.0060**	***P*** < **0.0001**
BCL6	*P* = 0.4579	*P* = 0.3122	*P* = 0.1254	*P* = 0.1545	*P* = 0.3369	*P* = 0.6179	*P* = 0.6223	*P* = 0.9521
MUM1	***P*** = **0.0026**	*P* = 0.9544	***P*** < **0.0001**	***P*** = **0.0001**	***P*** < **0.0001**	*P* = 0.6179	***P*** < **0.0001**	***P*** = **0.0005**

Abbreviations: LDH: serum lactate dehydrogenase; ECOG PS: Eastern Cooperative Oncology Group performance status; ESI: extranodal sites involvement; IPI: International prognostic index.

**Table 2:** The clinical characteristics between GCB and non-GCB group

Variables	Total no. (%)	GCB no.(%)	Non-GCB no.(%)	*P* value	Variables	Total no. (%)	GCB no.(%)	Non-GCB no. (%)	*P* value
**Age**	601	232	369		**ECOG PS**	597	229	368	
>60y	259 (43.1)	84 (36.2)	175 (47.4)	**0.0069**	0–1	484	208 (90.8)	276 (75)	**<0.0001**
≤60y	342 (56.9)	148 (63.8)	194 (52.6)		2–4	113	21 (9.2)	92 (25)	
**Sex**	601	232	369		**ESI**	600	231	369	
Male	345 (57.4)	131 (56.5)	214 (58.0)	0.7121	0–1	460	186 (80.5)	274 (74.3)	0.0775
Female	256 (42.6)	101 (43.5)	155 (42.0)		>1	140	45 (19.5)	95 (25.7)	
**LDH**	600	231	369		**IPI**	597	229	368	
Over ULN	302 (50.3)	84 (36.4)	218 (59.1)	**<0.0001**	0–2	398	179 (78.2)	219 (59.5)	**<0.0001**
Normal	298 (49.7)	147 (63.6)	151 (40.9)		>2	199	50 (21.8)	149 (40.5)	
**Stage**	600	231	369		**B symptom**	597	229	368	
I/II	226 (37.7)	116 (50.2)	110 (29.8)	**<0.0001**	Positive	218	56 (24.5)	162 (44.0)	**<0.0001**
III/IV	374 (62.3)	115 (49.8)	259 (70.2)		Negative	379	173 (75.5)	206 (56.0)	

Abbreviations: GCB: germinal center B-cell; LDH: serum lactate dehydrogenase; ULN: upper limit of normal; ECOG PS: Eastern Cooperative Oncology Group performance status; ESI: extranodal sites involvement; IPI: International prognostic index.

**Table 3:** The differences of clinical features between the DP and GCB* or non-GCB group.

Variables	Double positive no. (%)	Vs. GCB* no. (%)	*P*value	Vs. non-GCB no. (%)	*P*value
**Age**	80	152		369	
>60y	36 (45)	48 (31.6)	**0.0432**	175 (47.4)	0.6935
≤60y	44 (55)	104 (68.4)		194 (52.6)	
**Sex**	80	152		369	
Male	42 (52.5)	89 (58.6)	0.3768	214 (58.0)	0.3681
Female	38 (47.5)	63 (41.4)		155 (42.0)	
**LDH**	79	152		369	
Over ULN	30 (38.0)	54 (35.5)	0.7136	218 (59.1)	**0.0006**
Normal	49 (62.0)	98 (64.5)		151 (40.9)	
**Stage**	79	152		369	
I/II	39 (49.4)	77 (50.7)	0.8523	110 (29.8)	**0.0008**
III/IV	40 (50.6)	75 (49.3)		259 (70.2)	
**ECOG PS**	78	151		368	
0–1	66 (84.6)	142 (94.0)	**0.0192**	276 (75.0)	0.0681
2–4	12 (15.4)	9 (6.0)		92 (25.0)	
**ESI**	79	152		369	
0–1	62 (78.5)	124 (81.6)	0.5728	274 (74.3)	0.4311
>1	17 (21.5)	28 (18.4)		95 (25.7)	
**IPI**	78	151		368	
0–2	59 (75.6)	120 (79.5)	0.5062	219 (59.5)	**0.0076**
>2	19 (24.4)	31 (20.5)		149 (40.5)	
**B symptom**	78	151		368	
Positive	18 (23.1)	38 (25.2)	0.7275	162 (44.0)	**0.0006**
Negative	60 (76.9)	113 (74.8)		206 (56.0)	
**Myc expression**	29	70		148	
Positive	9 (31.0)	23 (32.9)	0.860	59 (39.9)	0.371
Negative	20 (69.0)	47 (67.1)		89 (60.1)	
**Bcl2 expression**	29	70		148	
Positive	12 (41.4)	29 (41.4)	0.996	90 (60.8)	0.053
Negative	17 (58.6)	41 (58.6)		58 (39.2)	
**DEL**	4 (13.8)	14 (20.0)	0.466	48 (32.4.)	**0.044**
Non-DEL	25 (86.2)	56 (80.0)		100 (67.6)	
***MYC*****-R**	28	70		148	
Positive	7 (25.0)	14 (20.0)	0.586	12 (8.1)	**0.016**
Negative	21 (75.0)	56 (80.0)		136 (91.9)	
***BCL*****2-R**	28	70		148	
Positive	1 (3.6)	14 (20.0)	0.060	15 (10.1)	0.474
Negative	27 (96.4)	56 (80.0)		133 (89.9)	
**DHL**	3 (10.7)	2 (2.9)	0.139	2 (1.4)	**0.029**
Non-DHL	25 (89.3)	68 (97.1)		146 (98.6)	

Abbreviations: GCB: germinal center B-cell; DP: double positive (CD10^+^MUM1^+^); LDH: serum lactate dehydrogenase; ULN: upper limit of normal; ECOG PS: Eastern Cooperative Oncology Group performance status; ESI: extranodal sites involvement; IPI: International prognostic index; DEL: double expression lymphoma; R: rearrangement; DHL: double hit lymphoma.

**Table 4:** The differences of clinical characteristics between the TN and non-GCB* or GCB group.

Variables	Triple negative no. (%)	Vs. non-GCB* no. (%)	*P*value	Vs. GCB no. (%)	*P*value
**Age**	53	316		232	
>60y	23 (43.4)	153 (48.4)	0.4982	84 (36.2)	0.3295
≤60y	30 (56.6)	163 (51.6)		148 (63.8)	
**Sex**	53	316		232	
Male	29 (54.7)	185 (58.5)	0.6014	131 (56.5)	0.8170
Female	24 (45.3)	131 (41.5)		101 (43.5)	
**LDH**	53	316		231	
Over ULN	25 (47.2)	193 (61.1)	0.0567	84 (36.4)	0.1446
Normal	28 (52.8)	123 (38.9)		147 (63.6)	
**Stage**	53	316		231	
I/II	22 (44.0)	88 (27.8)	**0.0442**	116 (50.2)	0.2527
III/IV	31 (56.0)	228 (72.2)		115 (49.8)	
**ECOG PS**	53	315		229	
0–1	46 (86.8)	230 (73.0)	**0.0321**	208 (90.8)	0.3758
2–4	7 (13.2)	85 (27.0)		21 (9.2)	
**ESI**	53	316		231	
0–1	43(81.1)	231 (73.1)	0.2159	186 (80.5)	0.9189
>1	10(18.9)	85 (26.9)		45 (19.5)	
**IPI**	53	315		229	
0–2	41 (77.4)	178 (56.5)	**0.0042**	179 (78.2)	0.8982
>2	12 (22.6)	137 (43.5)		50 (21.8)	
**B symptom**	53	315		229	
Positive	17 (32.1)	145 (46.0)	0.0582	56 (24.5)	0.2537
Negative	36 (67.9)	170 (54.0)		173 (75.5)	
**Myc expression**	21	127		99	
Positive	3 (14.3)	56 (44.1)	**0.010**	30 (30.3)	0.123
Negative	18 (85.7)	71 (55.9)		67 (69.7)	
**Bcl2 expression**	21	127		99	
Positive	13 (61.9)	87 (68.5)	0.887	41 (41.4)	0.086
Negative	8 (38.1)	40 (31.5)		58 (58.9)	
**DEL**	3 (14.3)	45 (35.4)	**0.055**	18 (18.4)	1.000
Non-DEL	18 (85.7)	82 (65.6)		81 (81.6)	
***MYC*****-R**	21	127		98	
Positive	2 (9.5)	10 (7.9)	0.680	21 (21.4)	0.360
Negative	19 (90.5)	117 (92.1)		77 (78.6)	
***BCL2*****-R**	21	127		98	
Positive	5 (23.8)	10 (7.9)	**0.041**	15 (15.3)	0.345
Negative	16 (76.2)	117 (92.1)		83 (84.7)	
**DHL**	1 (4.8)	1 (0.8)	0.256	5 (5.1)	1.000
Non-DHL	20 (95.2)	126 (99.2)		93 (94.9)	

Abbreviations: GCB: germinal center B-cell; TN: triple negative (CD10^−^Bcl6^−^MUM1^−^); LDH: serum lactate dehydrogenase; ULN: upper limit of normal; ECOG PS: Eastern Cooperative Oncology Group performance status; ESI: extranodal sites involvement; IPI: International prognostic index; DEL: double expression lymphoma; R: rearrangement; DHL: double hit lymphoma.

**Table 5:** Characteristics of patients treated with chemoimmunotherapy.

Variables	Patients no. (%)	Variables	Patients no. (%)
**Age**		>1	82 (26.8)
>60y	133 (43.5)	**IPI**	
≤60y	173 (56.5)	0–2	201 (65.7)
**Sex**		>2	105 (34.3)
Male	180 (58.8)	**Subtype**	
Female	126 (41.2)	GCB	120 (39.2)
**LDH**		Non-GCB	186 (60.8)
Over ULN	138 (45.1)	**Response**	
Normal	168 (54.9)	CR (u) /PR	244 (79.7%)
**Stage**		No response	62 (20.3)
I/II	118 (38.6)	**B symptom**	
III/IV	188 (61.4)	Positive	103 (33.7)
**ECOG PS**		Negative	203 (66.3)
0–1	62 (20.3)	**Treatment**	
2–4	244 (79.7)	R-CHOP	173 (56.5)
**ESI**		R-DA-EPOCH	86 (28.1)
0–1	224 (73.2)	R-CHOP-like^¶^	47 (15.4)

^¶^Cases who received multiple regimens because of the following events: disease progression, cardiotoxicity of doxorubicin, accompanied hemophagocytic syndrome and extremely poor ECOG PS. The R-CHOP-like regimens including R-CDOP, R-CEOP and R-mini-CHOP. Abbreviations: LDH: serum lactate dehydrogenase; ULN: upper limit of normal; ECOG PS: Eastern Cooperative Oncology Group performance status; ESI: extranodal sites involvement; IPI: International prognostic index; GCB: germinal center B-cell; CR (u): complete remission (unconfirmed); PR: partial remission.
