# Supplementary material for: The distinct clinical features and prognosis of the CD10+MUM1+ and CD10−Bcl6−MUM1− diffuse large B-cell lymphoma
Source: Sci Rep. 2016 Feb 9;6:20465. doi: 10.1038/srep20465 (PMC4746587; doi:10.1038/srep20465)
Supplement: Supplementary Information [file srep20465-s1.pdf]

Title

The distinct clinical features and prognosis of the $CD10^{+}MUM1^{+}$ and $CD10^{-}Bcl6^{-}MUM1^{-}$ diffuse large B-cell lymphoma

Name of authors

Ting-Xun Lu, Yi Miao, Jia-Zhu Wu, Qi-Xing Gong, Jin-Hua Liang, Zhen Wang, Li Wang, Lei Fan, Dong Hua, Yao-Yu Chen, Wei Xu, Zhi-Hong Zhang, Jian-Yong Li

Table S1. The clinical characteristics between the total cohort and the chemoimmunotherapy cohort

Variables	Total cohort no. (%)	R-CHOP-like cohort no. (%)	*P* value
**Age**	601	306	
> 60y	259 (43.1)	133 (43.5)	0.915
≤ 60y	342 (56.7)	173 (56.5)	
**Sex**	601	306	
Male	345 (57.4)	180 (58.8)	0.682
Female	256 (42.6)	126 (41.2)	
**LDH**	600	306	
Over ULN	302 (50.3)	138 (45.0)	0.136
Normal	298 (49.7)	168 (55.0)	
**Stage**	600	306	
I/II	226 (37.7)	118 (38.6)	0.793
III/IV	374 (62.3)	188 (61.4)	
**ECOG PS**	597	306	
0-1	484 (81.1)	244 (79.7)	0.631
2-4	113 (18.9)	62 (20.3)	
**ESI**	600	306	
0-1	460 (76.7)	224 (73.2)	0.252
> 1	140 (23.3)	82 (26.8)	
**IPI**	597	306	
0-2	398 (66.7)	201 (65.7)	0.768
> 2	199 (33.3)	105 (34.3)	
**B symptom**	597	306	
Positive	218 (36.5)	103 (33.7)	0.396
Negative	379 (63.5)	203 (66.3)	

Abbreviations: LDH: serum lactate dehydrogenase; ULN: upper limit of normal; ECOG PS: Eastern Cooperative Oncology Group performance status; ESI: more than one extranodal sites involvement; IPI: International prognostic index.

Table S2. The differences of clinical features of DP or TN group between the total cohort and the chemoimmunotherapy cohort

Variables	DP Total cohort no.(%)	DP R-CHOP-like cohort no.(%)	DP *P* value	TN Total cohort no.(%)	TN R-CHOP-like cohort no.(%)	TN *P* value
**Age**	80	49		53	30	
> 60y	36 (45)	22 (44.9)	0.991	23 (43.4)	13 (43.3)	0.996
≤ 60y	44 (55)	27 (55.1)		30 (56.6)	17 (56.7)	
**Sex**	80	49		53	30	
Male	42 (52.5)	25 (51.0)	0.870	29 (54.7)	17 (56.7)	0.864
Female	38 (47.5)	24 (49.0)		24 (45.3)	13 (43.3)	
**LDH**	79	49		53	30	
Over ULN	30 (38.0)	16 (32.7)	0.542	25 (47.2)	13 (43.3)	0.736
Normal	49 (62.0)	33 (67.3)		28 (52.8)	17 (56.7)	
**Stage**	79	49		53	30	
I/II	39 (49.4)	25 (51.0)	0.856	22 (44.0)	8 (26.7)	0.176
III/IV	40 (50.6)	24 (49.0)		31 (56.0)	22 (73.3)	
**ECOG PS**	78	49		53	30	

0-1	66 (84.6)	40 (81.6)	0.660	46 (86.8)	25 (83.3)	0.749
2-4	12 (15.4)	9 (18.4)		7 (13.2)	5 (16.7)	
**ESI**	79	49		53	30	
0-1	62 (78.5)	38 (77.6)	0.902	43(81.1)	22 (73.3)	0.408
> 1	17 (21.5)	11 (22.4)		10(18.9)	8 (26.6)	
**IPI**	78	49		53	30	
0-2	59 (75.6)	37 (75.5)	0.987	41 (77.4)	20 (66.7)	0.289
> 2	19 (24.4)	12 (24.5)		12 (22.6)	10 (33.3)	
**B symptom**	78	49		53	30	
Positive	18 (23.1)	11 (22.4)	0.935	17 (32.1)	12 (40.0)	0.467
Negative	60 (76.9)	38 (77.6)		36 (67.9)	18 (60.0)	

Abbreviations: GCB: germinal center B-cell; DP: double positive ($CD10^{+}MUM1^{+}$); LDH: serum lactate dehydrogenase; ULN: upper limit of normal; ECOG PS: Eastern Cooperative Oncology Group performance status; ESI: more than one extranodal sites involvement; IPI: International prognostic inde

Figure Legends

Figure S1. The immunohistochemical staining of the antibodies used in this study.

(A) CD10 positive. (B) CD10 negative. (C) MUM1 positive. (D) MUM1 negative. (E) Bcl6 positive. (F) Bcl6 negative. (G) Myc positive. (H) Myc negative. (I) Bcl2 positive. (J) Bcl2 negative.

Figure S2. FISH results of each probe

(A) *MYC*-R^+. (B) *MYC*-R^-. (C) *BCL2*-R^+. (D) *BCL2*-R^-.(E) *BCL6*-R^+. (F) *BCL6*-R^-.

Abbreviations: R: rearrangement; $^+$: positive; $^-$: negative.

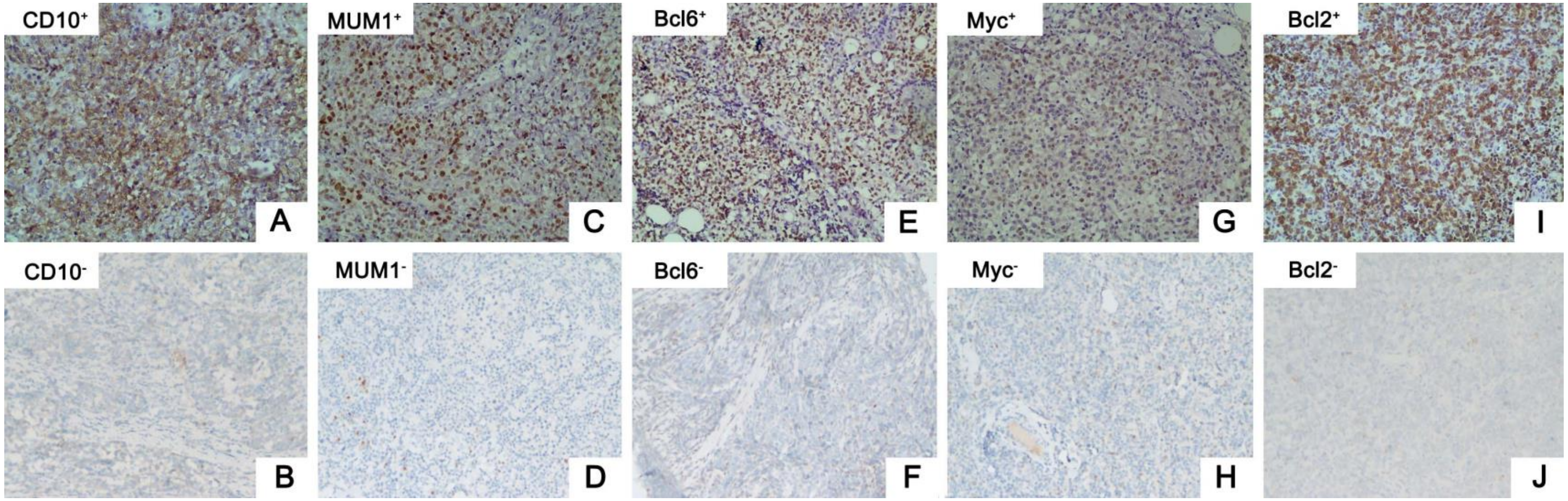
CD10+
A
MUM1+
C
Bcl6+
E
Myc+
G
Bcl2+
I
CD10-
B
MUM1-
D
Bcl6-
F
Myc-
H
Bcl2-
J

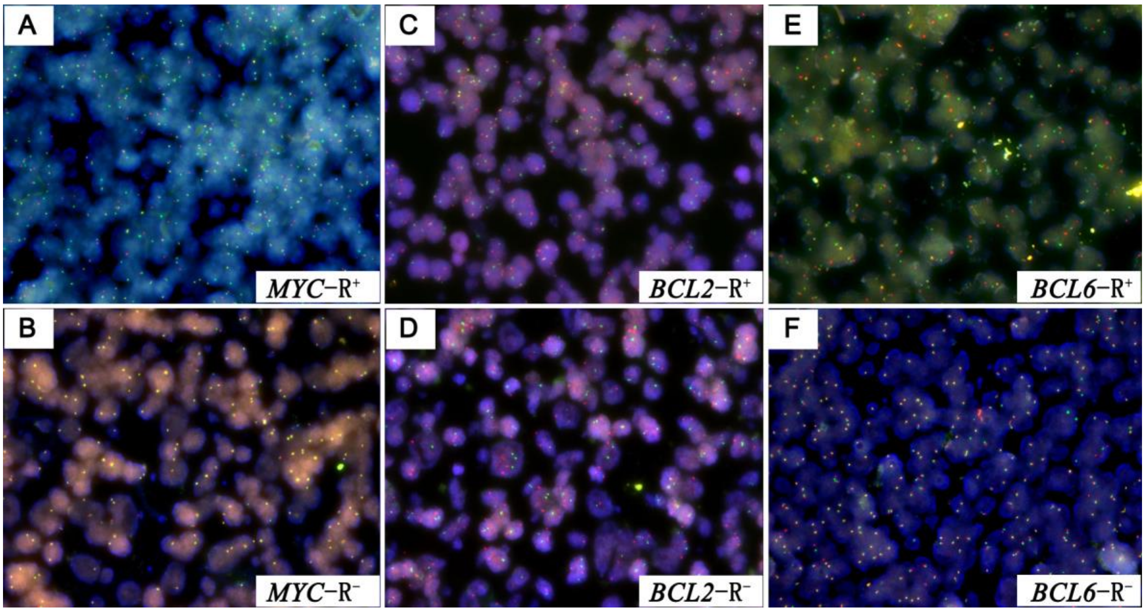
A
MYC-R+
C
BCL2-R+
E
BCL6-R+
B
MYC-R−
D
BCL2-R−
F
BCL6-R−
